# Obesity enhances nongenomic estrogen receptor crosstalk with the PI3K/Akt and MAPK pathways to promote *in vitro *measures of breast cancer progression

**DOI:** 10.1186/bcr3453

**Published:** 2013-07-23

**Authors:** Laura W Bowers, David A Cavazos, Ilane XF Maximo, Andrew J Brenner, Stephen D Hursting, Linda A deGraffenried

**Affiliations:** 1 Department of Nutritional Sciences, University of Texas at Austin - DPRI, 1400 Barbara Jordan Blvd, R1800, Austin, TX 78723-3092, USA; 2 Division of Hematology and Medical Oncology, University of Texas Health Science Center, 7703 Floyd Curl Drive, San Antonio, TX 78229, USA; 3 Department of Molecular Carcinogenesis, UT-MD Anderson Cancer Center Science Park, 1808 Park Road 1C, Smithville, TX 78957, USA; 4 Department of Cellular and Structural Biology, University of Texas Health Science Center, 7703 Floyd Curl Drive, San Antonio, TX 78229, USA

**Keywords:** obesity, breast cancer, estrogen receptor, Akt, MAPK, crosstalk

## Abstract

**Introduction:**

Epidemiological and clinical studies indicate that obesity is associated with a worse postmenopausal breast cancer prognosis and an increased risk of endocrine therapy resistance. However, the mechanisms mediating these effects remain poorly understood. Here we investigate the molecular pathways by which obesity-associated circulating factors in the blood enhance estrogen receptor alpha (ERα) positive breast cancer cell viability and growth.

**Methods:**

Blood serum was collected from postmenopausal breast cancer patients and pooled by body mass index (BMI) category (Control: 18.5 to 24.9 kg/m^2^; Obese: ≥30.0 kg/m^2^). The effects of patient sera on MCF-7 and T47D breast cancer cell viability and growth were examined by MTT and colony formation assays, respectively. Insulin-like growth factor receptor 1(IGF-1R), Akt, and ERK1/2 activation and genomic ERα activity were assessed to determine their possible contribution to obese patient sera-induced cell viability and growth. To further define the relative contribution of these signaling pathways, cells grown in patient sera were treated with various combinations of ERα, PI3K/Akt and MAPK targeted therapies. Comparisons between cells exposed to different experimental conditions were made using one-way analysis of variance (ANOVA) and Student's *t *test.

**Results:**

Cells grown in media supplemented with obese patient sera displayed greater cell viability and growth as well as IGF-1R, Akt and ERK1/2 activation relative to control sera. Despite the lack of a significant difference in genomic ERα activity following growth in obese versus control patient sera, we observed a dramatic reduction in cell viability and growth after concurrent inhibition of the ERα and PI3K/Akt signaling pathways. Further, we demonstrated that ERα inhibition was sufficient to attenuate obese serum-induced Akt and ERK1/2 activation. Together, these data suggest that obesity promotes greater ERα positive breast cancer cell viability and growth through enhanced crosstalk between nongenomic ERα signaling and the PI3K/Akt and MAPK pathways.

**Conclusions:**

Circulating factors in the serum of obese postmenopausal women stimulate ERα positive breast cancer cell viability and growth by facilitating non-genomic ERα crosstalk with the PI3K/Akt and MAPK signaling pathways. These findings provide valuable insight into one mechanism by which obesity may promote ERα positive postmenopausal breast cancer progression and endocrine therapy resistance.

## Introduction

The prevalence of obesity in the United States has been climbing steadily for the past three decades, resulting in a current adult obesity rate of 35.7% [1]. A similar trend is evident in other nations around the world and is no longer unique to wealthy, industrialized countries [2]. This epidemic poses a dire threat to public health, as obesity can play a role in the pathogenesis of numerous diseases, including breast cancer. In postmenopausal women, obesity increases breast cancer risk by approximately 40% [3-5]. A large body of evidence has also established that obesity is associated with a worse breast cancer prognosis for both pre- and postmenopausal women. One prospective study that followed a population of more than 900,000 US adults over a 16-year period found that the mortality rate due to breast cancer was amplified with each successive increase in body mass index (BMI) category [6]. Another study showed a significantly greater risk for disease recurrence within 10 years of diagnosis in breast cancer patients who were obese at the time of treatment in comparison to non-obese patients [7]. These effects could be due to later diagnosis in the obese population, resulting in more advanced disease at the time of diagnosis. This hypothesis was initially supported by data from a large cohort of patients followed for a 20-year period; Majed *et al*. [8] found that the obese patients presented with more advanced tumors, suggesting that diagnosis had been delayed. However, the authors ultimately found that multivariate analysis demonstrated an independent effect of obesity on breast cancer prognosis, regardless of tumor stage at time of diagnosis. Survival analysis revealed increased metastatic recurrence as well as decreased disease-free interval and overall survival in the obese patient population. While obesity has been shown to impact prognosis negatively for both pre- and postmenopausal patients, the most prominent effects are seen in estrogen receptor alpha (ERα) positive postmenopausal patients, a finding confirmed by a recent retrospective analysis of the German BRENDA-cohort [9].

Previous studies indicate that obesity may adversely impact prognosis in the ERα positive postmenopausal patient population in part by promoting endocrine therapy resistance [10]. This theory is supported by an analysis of data from the Arimidex, Tamoxifen Alone or in Combination (ATAC) trial by Sestak *et al*. [11], which found that obese breast cancer patients receiving anastrozole had a significantly greater risk of recurrence. In agreement with these findings, Schmid *et al*. [12] demonstrated that obese patients have a significantly reduced response rate to letrozole in comparison to lean (11% versus 35%). The ATAC trial also showed that while anatrozole treatment resulted in significantly greater recurrence-free survival in comparison to tamoxifen, this benefit was lost in the obese cohort [11]. The primary site of aromatase expression and estrogen production in postmenopausal women is the adipose tissue. Due to an abundance of this aromatase-expressing tissue, obese postmenopausal women typically have higher levels of circulating estradiol [13-15], and researchers have posited that this may contribute to the observed increase in breast cancer risk and worse outcome in this population. This hypothesis suggests that an adjustment of the aromatase inhibitor dosage may improve obese patient prognosis. However, that conclusion is confounded by two phase III clinical trials of anastrozole that found no overall benefit from a 10 mg dose (versus 1 mg), indicating that an increased dosage may not be effective in overcoming obesity-induced resistance to aromatase inhibitors [16,17].

The development of endocrine therapy resistance can be mediated by several mechanisms. Frequently, aberrant signaling from growth factor receptors, particularly the insulin-like growth factor 1 receptor (IGF-1R) and the HER family of receptors, is responsible. These receptors can engage in bidirectional crosstalk with ERα, leading to increased nongenomic ERα activity, ligand-independent activation of ERα, and abnormal regulation of cell cycle and apoptotic signaling. Nongenomic ERα activity results in the activation of the MAPK and PI3K/Akt signaling pathways, and these can in turn activate ERα via phosphorylation, leading to enhanced genomic ERα activity [18,19]. Obesity is typically accompanied by elevated circulating levels of insulin, bioavailable IGF-1 and leptin, as well as a series of pro-inflammatory cytokines [20-23]. All of these obesity-associated circulating factors are able to activate the PI3K/Akt and/or MAPK pathways, potentially enhancing the ERα crosstalk pathways described above and leading to endocrine resistance and breast cancer progression [24-28]. The metabolic alterations associated with obesity, including changes in insulin and insulin-like growth factor binding protein 1 (IGFBP-1) serum levels (which result in increased circulating free IGF-1 levels), are also significantly correlated with breast cancer recurrence and mortality [29]. High serum concentrations of pro-inflammatory cytokines and leptin have been similarly linked to a worse breast cancer outcome [30-32]. Overall, obesity creates a complex metabolic imbalance accompanied by chronic inflammation, enriching the blood with a number of signaling molecules that may promote breast cancer progression and adversely affect outcome.

This study utilized an *in vitro *model of obesity in which ERα positive breast cancer cells were exposed to pooled sera samples from normal weight or obese postmenopausal breast cancer patients. This model enabled us to examine the molecular pathways by which obesity-associated circulating factors in the blood stimulate greater ERα positive breast cancer cell viability and growth. Here we provide evidence that these physiological effects are mediated by enhanced crosstalk between nongenomic ERα signaling and the PI3K/Akt and MAPK pathways. These studies provide insight into one potential mechanism by which obesity may promote postmenopausal ERα positive breast cancer progression and endocrine therapy resistance.

## Methods

### Serum samples

Serum was collected from postmenopausal breast cancer patients under an Institutional Review Board (IRB) approved biorepository collection protocol at the Cancer Therapy and Research Center of the University of Texas Health Science Center at San Antonio (UTHSCSA). The collection and use of these biological samples was approved by the IRB of UTHSCSA (HSC20070684H) and conducted in accordance with the Declaration of Helsinki and good clinical practice. Informed consent was obtained prior to participation, and all samples and data were deidentified prior to release to maintain patient confidentiality. Serum was pooled according to the BMI category of the patient (normal weight (control): 18.5 to 24.9 kg/m^2^; obese: ≥30 kg/m^2^). The free IGF-1 concentration of each patient's serum sample was measured using the MILLIPLEX MAP Human IGF-1 Single Plex Metabolism Assay, while the MILLIPLEX MAP Human Serum Adipokine Panel A and B kits were used to assess patient serum concentrations of insulin, IL-6, TNFα, leptin, and adiponectin (EMD Millipore, Billerica, MA, USA).

### Cell lines and reagents

ERα positive MCF-7 and T47D cells (ATCC, Manassas, VA, USA) were maintained in improved minimum essential medium (IMEM) (GIBCO Life Technologies, Grand Island, NY, USA) supplemented with 10% fetal bovine serum (FBS). 3-(4,5-Dimethylthiazol-2-yl)-2,5-diphenyltetrazolium bromide (MTT reagent) was purchased from Sigma-Aldrich (St. Louis, MO, USA). The drug treatments used in this study, which include PD 98,059 (a MEK1 inhibitor), LY 294,002 (a PI3K inhibitor), and 4-hydroxytamoxifen (a selective estrogen receptor modulator), were also obtained from Sigma-Aldrich. The primary antibodies for pAkt (s473), tAkt, pERK1/2, tERK1/2, pERα (s167), pERα (s118), pIGF-1R (tyr1135/1136) and tIGF-1R were purchased from Cell Signaling (Beverly, MA, USA). The tERα primary antibody was produced by Novacastra (Leica Microsystems, Buffalo Grove, IL, USA).

### MTT assay

MCF-7 and T47D cells were seeded in IMEM supplemented with 10% FBS at a density of 8 × 10^3 ^in 96-well plates. After 24 hours of growth in the 10% FBS media, the cells were exposed to 2% sera in serum-free media (SFM), with or without the addition of drug treatments, for 48 hours. MTT reagent in PBS (5 mg/ml) was then added to each well to a final concentration of 0.5 mg/ml. After two hours of incubation at 37°C, the media were removed and 50 ul dimethyl sulfoxide (DMSO) added to each well to lyse the cells. Absorbance was read at 570 nm on a FLUOstar Omega Spectrometer (BMG Labtech, Offenberg, Germany). Relative cell viability was calculated by dividing each absorbance value by the absorbance for cells grown in control patient sera. Data shown represent the average of at least three independent experiments.

### Colony formation assay

MCF-7 and T47D cells were seeded in IMEM supplemented with 10% FBS at a density of 500 and 1 × 1 0^3^, respectively, in six-well plates. After 24 hours of growth in the 10% FBS media, the cells were continuously exposed to 2% sera in SFM, with or without drug treatments, for nine days. On day five of the treatment period, the wells were aspirated and washed, and the media were replenished with the same concentration of sera and inhibitors. On day nine, the colonies were fixed and stained with 1% crystal violet in 70% acetic acid for 30 minutes and then counted. The relative number of colonies, a reflection of cell growth, was calculated by dividing each colony count by the count for cells grown in control patient sera and multiplying by 100. Data shown represent the average of at least three independent experiments.

### Western blot analysis

The cells were grown to 80% confluence in IMEM supplemented with 10% FBS, then the growth medium was aspirated, the wells were washed, and the medium replaced with SFM overnight. After overnight serum-starvation to minimize the effect of growth factors and hormones in the growth medium, 2% obese or control patient serum was added directly to the overnight SFM with or without inhibitors for 15 minutes or one hour. Kinase lysis buffer or radioimmunoprecipitation assay (RIPA) buffer was used for protein extraction. Protein content of the lysates was measured using the BCA Protein Assay kit from Thermo Scientific Pierce (Rockford, IL, USA) or the Bio-Rad Protein Assay (Bio-Rad, Hercules, CA, USA). Images were acquired using a Syngene G:BOX Chemi (Frederick, MD, USA). Relative protein levels were calculated by first standardizing phosphorylated protein to total protein levels for each experimental condition, then dividing the standardized protein level for each condition by that of cells grown in control patient sera. Data from at least three independent experiments were compiled for each protein and cell line to calculate the average protein level, standard error of the mean and statistical significance, with one representative image for each protein shown.

### Estrogen response element luciferase assay

A luciferase reporter gene driven by a 3X estrogen response element (ERE)-tk promoter (a kind gift from Dr. Arun Roy, UTHSCSA) was utilized to measure ERα transcriptional activity. Transient transfections were performed in triplicate wells three times. MCF-7 and T47D cell lines were seeded in IMEM supplemented with 10% FBS at a density of 1.5 × 10^4 ^in 24-well plates and concurrently transfected with the ERE luciferase and renilla plasmids after 24 hours of growth using Fugene 6 from Promega (Madison, WI, USA) at a 1:3 ratio. The cells were serum-starved for six hours the following day, then exposed for 48 hours to 2% obese or control patient serum, added directly to the SFM. Luciferase activity was then measured using Promega's Dual Luciferase Reporter Assay System, with the fluorescence read on a FLUOstar Omega Spectrometer (BMG Labtech, Offenberg, Germany). Relative ERα activity was calculated by dividing the fluorescence value (standardized to renilla) from cells grown in obese patient sera by that from cells grown in control patient sera. Data shown represent the average of at least three independent experiments.

### Quantitative RT-PCR

Total RNA was isolated using TRIzol reagent (Invitrogen) and reverse transcribed with Promega's ImProm II Reverse Transcription System. The primer sequences are as follows: pS2: forward, 5'-GGTCGCCTTGGAGCAGA-3'; reverse, 5'-GGGCGAAGATCACCTTGTT-3'; cyclin D1: forward, 5'-TGGAGGTCTGCGAGGAACAGAA-3'; reverse, 5'-TGCAGGCGGCTCTTTTTCA-3'. The manufacturer's recommended cycling conditions for the QuantiFast SYBR Green PCR kit (Qiagen) were used. Data shown represent the average of at least three independent experiments.

### Statistics

Differences between cells exposed to obese versus control sera were measured using Student's *t *test. One-way analysis of variance (ANOVA) was used to analyze differences between more than two experimental conditions. A difference of *P *< 0.05 was considered significant.

## Results

### Patient characteristics

Table 1 describes the postmenopausal breast cancer patients who provided the sera utilized in this study, which was pooled into two groups by BMI category, obese (Ob) and control (Con). There was no significant difference in the average patient age between the groups. The average patient BMI in the Ob group was significantly higher than the Con group (*P *< 0.01), and this was accompanied by significantly higher levels of IL-6 (*P *< 0.05), TNFα (*P *< 0.05), and leptin (*P *< 0.01), as well as lower levels of adiponectin (*P *< 0.05). In addition, the Ob group's serum insulin levels were almost five-fold greater, a difference that approached significance (*P *= 0.10), but there was no difference in free IGF-1 concentration. Sixty percent of the patients in the obese group were Hispanic, while the control group was predominantly white. Diabetes and hypercholesterolemia were found in at least 25% of the patients in the obese group, but were not present in the control group. Those diagnosed with these conditions had all been prescribed metformin and statins, respectively, two drugs with possible anti-cancer effects (33-38). The majority of patients in both groups were receiving either aromatase inhibitor or tamoxifen treatment.

**Table 1:** Patient characteristics

	Obese	Control
Number of patients	20	5
Average age (years)	59.7 (6.28)	59.5 (4.76)
Average BMI (kg/m^2^)	36.6 (5.04)**	21.1 (2.17)
Average serum concentrations		
Free IGF-1 (ng/ml)	4.92 (0.925)	5.43 (1.80)
Insulin (pg/ml)	742.9 (156.8)	157.4 (44.1)
Interleukin 6 (pg/ml)	4.6 (0.58)*	1.6 (0.29)
TNF-α (pg/ml)	7.1 (0.51)*	4.6 (0.19)
Leptin (ng/ml)	36.6 (4.67)**	6.40 (3.86)
Adiponectin (ug/ml)	54.5 (6.55)*	97.5 (32.0)
Ethnicity/race		
Hispanic	12	0
White	6	3
African-American	2	0
Asian	0	1
Not Available	0	1
Confounding conditions		
Diabetes	5	0
Hypercholesterolemia	8	0
Hypertension	14	1
Medications		
Aromatase inhibitor	9	1
Tamoxifen	5	2
Chemotherapy	6	2
Metformin	5	0
Statin	8	0

(Standard error of the mean); *, *P *< 0.05; **, *P *< 0.01 in comparison to control. BMI, body mass index; IGF-1, insulin-like growth factor 1; TNF-α, tumor necrosis factor- alpha.

### Obesity-associated circulating factors enhance breast cancer cell viability and growth

**Figure 1:**
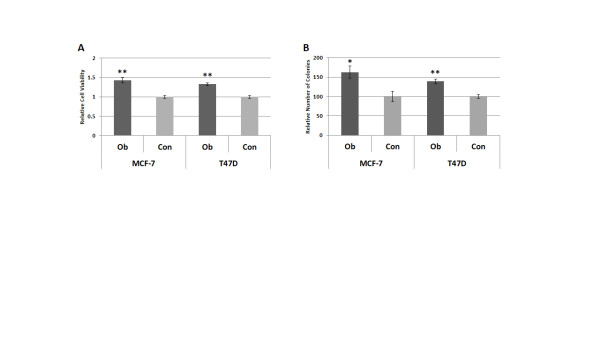
**Obesity-associated circulating factors promote greater breast cancer cell viability and growth**. (**A**) MCF-7 and T47D breast cancer cells were exposed to 2% obese (Ob) or control (Con) patient sera for 48 hours; viability was then measured by MTT assay. (**B**) Colony formation assay was utilized to assess MCF-7 and T47D cell growth following a nine day exposure to 2% Ob or Con patient sera. Data shown represent the average of at least three independent experiments. *, *P *< 0.05; **, *P *< 0.01 in comparison to Con. MTT reagent, 3-(4,5-dmethylthiazol-2-yl)-2,5-diphenyltetrazolium bromide.

In order to elucidate the potential mechanisms by which obesity promotes breast cancer progression, we first evaluated the effect of obesity-associated circulating factors on cell viability and growth, both *in vitro *parameters of cancer aggression. Viability of breast cancer cells in response to exposure to patient serum was measured by MTT assay. MCF-7 cells grown in 2% Ob sera in SFM for 48 hours displayed a 43% increase in viability in comparison to cells grown in Con sera (*P *< 0.01). Ob sera also enhanced the viability of T47D cells by 32% versus Con sera (*P *< 0.01) (Figure 1A). Colony formation assay was utilized to assess the effects of patient sera on breast cancer cell growth. Both MCF-7 and T47D cells grew significantly better in Ob sera, forming 63% (*P *< 0.05) and 39% (*P *< 0.01) more colonies, respectively, over a nine day exposure to the Ob sera in comparison to Con sera (Figure 1B). These results demonstrate that one or more circulating factors in the obese patient sera directly induces higher levels of ERα positive breast cancer cell viability and growth.

### PI3K/Akt, MAPK, and IGF-1R pathway activation is stimulated by obesity-associated circulating factors in breast cancer cells

**Figure 2:**
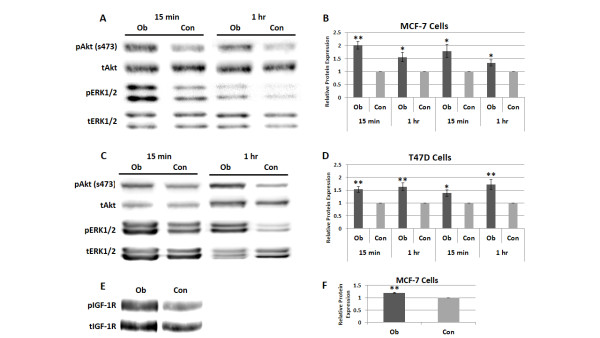
**Breast cancer cell Akt, ERK1/2, and IGF-1R activation are enhanced by obesity-associated circulating factors**. The effects of obese (Ob) and control (Con) patient sera on Akt, ERK1/2 and IGF-1R activation were assessed by western blotting. MCF-7 (**A **and **B**) and T47D (**C **and **D**) cells were exposed to 2% Ob or Con patient sera for 15 minutes or one hour; pAkt (ser473) and pERK1/2 protein levels were then measured and standardized to tAkt and tERK1/2 protein levels, respectively. (**E **and **F**) pIGF-1R (tyr1135/1136) protein levels in MCF-7 cells were measured and standardized to tIGF-1R following a 15 minute incubation in 2% Ob or Con patient sera. Densitometry data from at least three independent experiments were compiled for each protein and cell line to calculate the average protein level, standard error of the mean and statistical significance, with one representative image for each protein shown. *, *P *< 0.05; **, *P *< 0.01 in comparison to Con. IGF-1R, insulin-like growth factor 1 receptor.

The PI3K/Akt and MAPK pathways are both downstream targets common to many of the circulating factors typically upregulated with obesity [24-28]. They are also involved in the regulation of cell proliferation and survival and can crosstalk with and ultimately activate ERα independent of estradiol [18,19]. Consequently, we assessed the effects of Ob and Con sera on Akt and ERK1/2 activation. MCF-7 cells exposed to 2% Ob sera for 15 minutes or one hour had 100% (*P *< 0.01) and 55% (*P *< 0.05) higher levels of pAkt (ser473), respectively, in comparison to cells exposed to Con sera. pERK1/2 levels following 2% Ob sera exposure were 79% and 33% (*P *< 0.05) greater at the same time points in comparison to Con (Figures 2A and 2B). A similar effect was observed in T47D cells exposed to Ob versus Con sera at these time points. Ob sera exposure stimulated 53% and 64% (*P *< 0.01) more Akt activation and 38% (*P *< 0.05) and 72% (*P *< 0.01) more ERK1/2 activation than Con after 15 minute or 1 hour incubation periods, respectively (Figures 2C and 2D). These results suggest that the PI3K/Akt and MAPK pathways may both play a role in obesity-induced breast cancer progression. Despite finding no difference between the two patient groups in average serum free IGF-1, MCF-7 cells exposed to the Ob sera had 20% (*P *< 0.01) higher pIGF-1R (tyr1135/1136) levels in comparison to Con (Figures 2E and 2F). This suggests that the Ob sera-induced Akt and ERK1/2 activation described above may be at least partly mediated by IGF-1R signaling that is upregulated by a mechanism independent of bioavailable IGF-1 levels.

### Genomic ERα activity in breast cancer cells is not directly enhanced by obesity-associated circulating factors

**Figure 3:**
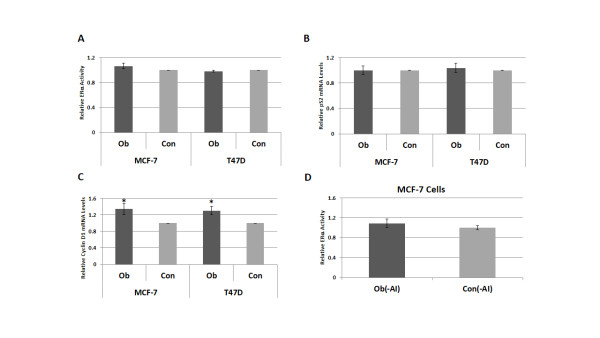
**Genomic ERα activity in breast cancer cells is not directly enhanced by obesity-associated circulating factors**. Genomic ERα activity in response to 2% obese (Ob) or control (Con) patient sera exposure was measured in MCF-7 and T47D cells with an ERE luciferase reporter (**A**) and qPCR analysis of pS2 expression (**B**). Expression of cyclin D1, which is regulated by both ERα and the PI3K/Akt and MAPK pathways, was assessed by qPCR analysis in both cell lines following growth in 2% Ob or Con patient sera (**C**). The effect of 2% Ob(-AI) and Con(-AI) patient sera on genomic ERα activity in MCF-7 cells was also measured by ERE luciferase reporter (**D**). This pooled sera excluded breast cancer patients receiving aromatase inhibitors at the time of collection. Data shown represent the average of at least three independent experiments. *, *P *< 0.05 in comparison to Con. ERα, estrogen receptor alpha; ERE, estrogen response element.

In addition to an elevation in circulating levels of growth factors, pro-inflammatory cytokines, and leptin, obesity in postmenopausal women is typically accompanied by higher levels of circulating estrogens [39]. In ERα positive breast cancer cells, estradiol can bind ERα and activate its canonical signaling pathway, in which ERα acts as a nuclear transcription factor or cofactor, modulating the expression of its target genes in a manner that promotes cell proliferation and growth. This genomic ERα activity can also be induced via ligand-independent phosphorylation of the receptor's AF-1 domain by Akt and ERK1/2 [18,19,40]. To assess the effect of Ob and Con sera on genomic ERα activity, we measured relative ERE luciferase reporter activity in MCF-7 and T47D cells in response to these conditions. No significant difference was detected in the luciferase activity, suggesting that the factors in Ob sera do not directly enhance genomic ERα activity (Figure 3A). Expression of pS2, an ERα target gene, was also measured as another indicator of ERα transcriptional activity. qPCR analysis of the relative levels of pS2 mRNA showed no difference in pS2 expression in either the MCF-7 or T47D cell lines after growth in Ob versus Con sera (Figure 3B). In contrast, Ob sera did induce significantly higher expression of cyclin D1, another ERα target gene, in both cell lines. MCF-7 cells expressed 34% more cyclin D1 following 24 hours of growth in Ob sera versus Con, while cyclin D1 mRNA levels were 30% higher in T47D cells under these conditions (*P *< 0.05) (Figure 3C). However, while pS2 expression is considered to be a very specific and reliable indicator of ERα activity, cyclin D1 expression is regulated by many signaling pathways, including PI3K/Akt and MAPK. Therefore, the upregulation of cyclin D1 expression following Ob sera exposure is likely related to increased activity in these upstream pathways. Because cyclin D1 is involved in promoting progression through the cell cycle, these results are also supportive of our data demonstrating a significant difference in breast cancer cell growth following Ob sera exposure.

One potential critique of our study design is the use of sera from breast cancer patients. Many of the patients who provided sera for this study were receiving aromatase inhibitor treatment at the time of serum collection, leading to a decrease in their circulating estradiol levels. The lack of difference in genomic ERα activity could be an artifact of the drug's effects. To address this issue, we repeated the ERE luciferase assay in MCF-7 cells with pooled sera from patients who had not been prescribed aromatase inhibitors (Ob(-AI) versus Con(-AI)) and again found no difference in genomic ERα activity (Figure 3D). Together, these studies strongly suggest that genomic ERα activity plays a minimal role in mediating obese sera-induced breast cancer cell viability and growth.

### Combined PI3K and ERα inhibition attenuates effects of obese patient sera on breast cancer cell viability and growth

**Figure 4:**
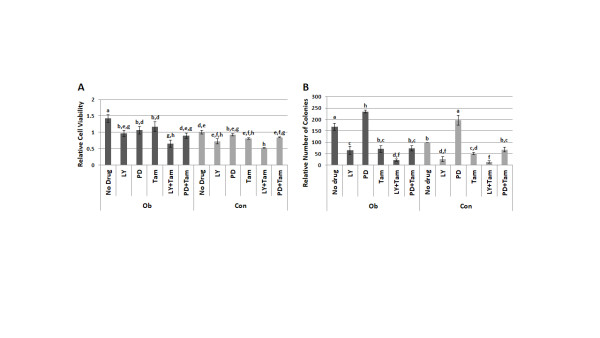
**Combined PI3K/ERα inhibition attenuates effects of obesity on breast cancer cell viability and growth**. The contribution of the PI3K/Akt, MAPK, and ERα pathways to obese (Ob) patient sera-induced cell viability and growth was examined via treatment of MCF-7 cells with the following inhibitors during sera exposure: LY 294,002 (LY, a PI3K inhibitor, 10uM), PD 98,059 (PD, a MEK1 inhibitor, 10uM) and 4-hydroxytamoxifen (Tam, a selective estrogen receptor modulator, 100 nM). (**A**) MCF-7 cell viability was measured by MTT assay following a 48 hour exposure to 2% Ob or control (Con) patient sera, with or without drug treatment. (**B**) Colony formation assay was used to assess MCF-7 cell growth over a nine day exposure to 2% Ob or Con patient sera, with or without drug treatment. Data shown represent the average of at least three independent experiments, and different letters indicate significant differences (*P *< 0.05). ERα, estrogen receptor alpha; MTT reagent, 3-(4,5-dmethylthiazol-2-yl)-2,5-diphenyltetrazolium bromide.

After demonstrating that Ob sera exposure directly increases PI3K/Akt and MAPK pathway activation, but not genomic ERα activity, we examined the contribution of these pathways to Ob sera-induced MCF-7 cell viability and growth. Using the targeted inhibitors LY 294,002 (LY, a PI3K inhibitor), PD 98,059 (PD, a MEK1 inhibitor) and 4-hydroxytamoxifen (Tam, a selective estrogen receptor modulator), we established which factors were essential for the observed increase in viability and growth. While each drug was able to significantly decrease the viability of MCF-7 cells exposed to Ob sera (*P *< 0.05), LY/Tam inhibited viability by 54% and was the only treatment able to inhibit it to a level significantly less than cells grown in Con sera (*P *< 0.05). In addition, cells exposed to Con sera and LY/Tam had a significantly lower viability level in comparison to all Ob sera-exposed cells (*P *< 0.05) except those also treated with LY/Tam, suggesting that this drug combination is the most effective at neutralizing obesity-induced viability (Figure 4A). Ob sera-induced MCF-7 cell growth was significantly decreased by all drug treatments except PD. However, the LY/Tam combination again proved to be the most effective inhibitor; it decreased Ob sera-induced growth by 87%, inhibiting it to a level significantly lower than that produced by all other drug treatments (*P *< 0.01). Intriguingly, PD alone significantly increased the number of colonies formed by MCF-7 cells grown in Ob or Con sera, but also inhibited Ob sera-induced growth when administered in combination with Tam (*P *< 0.01) (Figure 4B). These results suggest that signaling from all three pathways, as well as enhanced crosstalk between them, contributes to the upregulation of breast cancer cell viability and growth by obese patient sera. However, because the most effective drug combination was LY/Tam, the data also indicates that the PI3K/Akt pathway and its interactions with ERα may play a more critical role than the MAPK pathway in mediating these effects.

### Obesity-associated circulating factors enhance Akt-mediated activation of ERα and nongenomic ERα activity

**Figure 5:**
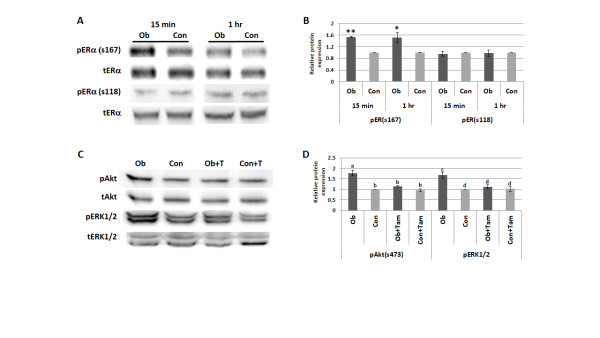
**Obesity-associated circulating factors promote greater Akt-mediated ERα phosphorylation and nongenomic ERα activity**. Phosphorylation of ERα at two different sites (ser167 and ser118, the Akt and MAPK target sites, respectively) following a 15 minute or one hour exposure to 2% obese (Ob) or control (Con) patient sera was assessed in MCF-7 cells by western blot and standardized to tERα protein levels (**A **and **B**). The effect of tamoxifen (T) treatment on Akt and ERK1/2 activation in MCF-7 cells following a 15 minute exposure to 2% Ob or Con patient serum was also measured by western blot (**C **and **D**). Densitometry data from at least three independent experiments was compiled for each protein to calculate the average protein level, standard error of the mean and statistical significance, with one representative image for each protein shown. *, *P *< 0.05; **, *P *< 0.01 in comparison to Con, and different letters indicate significant differences (*P *< 0.05). ERα, estrogen receptor alpha.

In addition to its transcriptional activity, ERα signaling also occurs at the plasma membrane and in the cytoplasm. Here, ERα can activate the PI3K/Akt and MAPK pathways when it forms complexes with other signaling molecules, including the IGF-1R and the regulatory subunit of PI3K, p85. Akt and ERK1/2 can in turn activate ERα in a ligand-independent manner by phosphorylation [18,19]. Although there was no difference in genomic ERα activity following Ob versus Con sera exposure, our data demonstrated that LY/Tam is the most effective drug combination for the inhibition of Ob sera-induced breast cancer cell viability and growth, indicating that ERα is indeed a critical player in mediating these effects. Consequently, we next examined whether nongenomic ERα activity is enhanced by obesity-associated circulating factors. We found that Ob sera, in comparison to Con, promotes 53% (*P *< 0.01) and 52% (*P *< 0.05) higher levels of ERα phosphorylation at the Akt target site (s167) in MCF-7 cells following a 15 minute or one hour exposure, respectively (*P *< 0.05). No difference between Ob and Con was seen at the ERK1/2 target site (ser118) under the same conditions (Figures 5A and 5B). Ob sera also stimulated an increase in Akt and ERK1/2 phosphorylation via ERα activity in the cytoplasm. This is demonstrated by the ability of Tam to inhibit Ob sera-induced Akt and ERK1/2 activation in MCF-7 cells by 36% (*P *< 0.01) and 33% (*P *< 0.05), respectively. In contrast, Tam had no effect on Con sera-induced Akt and ERK1/2 activation (Figures 5C and 5D). ERα inhibition also eliminated the difference in Akt and ERK1/2 activation levels stimulated by Ob and Con sera exposure alone, suggesting that obesity-associated circulating factors are promoting greater nongenomic ERα activity. This enhanced crosstalk explains why the addition of Tam to either LY or PD results in greater inhibition of Ob sera-induced breast cancer cell viability and growth in comparison to either drug alone.

## Discussion

Growth factor signaling is known to promote the development of endocrine resistance in breast cancer. However, while obesity has been shown to modulate growth factor signaling pathways, its impact on hormone independence remains relatively unexplored. We have previously reported that obese ovariectomized mice implanted with syngeneic mouse mammary tumor cells displayed enhanced mammary tumor development and progression, and this was associated with elevated levels of bioavailable IGF-1 and downstream PI3K/Akt/mTOR signaling [41,42]. Because elevated growth factor signaling can stimulate cytoplasmic ERα localization and nongenomic ERα activity [18], we investigated the role of bidirectional crosstalk among various growth factor pathways and ERα. Based on our current findings, we propose that obesity-induced systemic factors promote breast cancer progression and may increase resistance to aromatase inhibitor therapy by initiating crosstalk between nongenomic ERα activity and the IGF-1R, PI3K/Akt and MAPK signaling pathways.

Here we demonstrate that circulating factors associated with postmenopausal obesity increased ERα positive breast cancer cell viability and growth (Figure 1). This was coupled with greater breast cancer cell Akt and ERK1/2 phosphorylation, as well as enhanced IGF-1R activation (Figure 2). Intriguingly, there was no difference between the obese and control patients in average serum free IGF-1 concentration. However, average insulin levels were non-significantly higher in the obese group, and insulin can also bind and activate the IGF-1R (Table 1). The lack of significant differences in these hormones may be due to the non-fasting status of the patients, as other studies examining their association with obesity have assessed fasting serum samples [20,21]. Obese postmenopausal women are also known to have, on average, higher levels of circulating estradiol [13-15]. Consequently, we were surprised to find no difference in the genomic ERα activity of breast cancer cells grown in obese versus control patient sera, even with the exclusion of patients on aromatase inhibitors at the time of serum collection, suggesting that obesity-related circulating factors promote ERα positive breast cancer cell viability and growth independent of ERα transcriptional activity.

**Figure 6:**
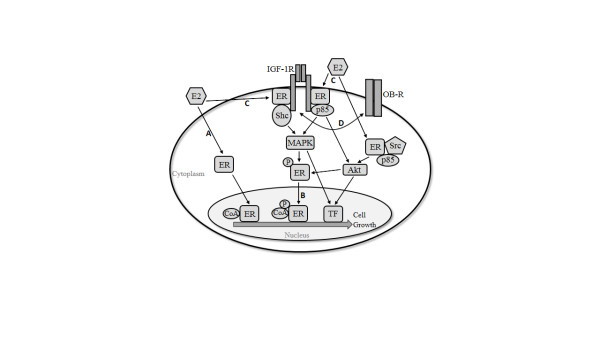
**Breast cancer cell estrogen receptor activity**. Three general mechanisms of ERα signaling are depicted. First, ERα can bind estradiol (E2) and enter the nucleus, where it forms complexes with co-activators (CoAs) and regulates gene transcription by binding directly to estrogen response elements (EREs) specific to ERα or via interaction with other transcription factors (**A**). This genomic ERα activity can also be stimulated by Akt or MAPK-mediated phosphorylation of the receptor (**B**). Ligand-bound ERα can also remain outside the nucleus, where it complexes with the IGF-1R at the cell membrane or with other signaling molecules in the cytoplasm to activate the PI3K/Akt and MAPK pathways (C). IGF-1R crosstalk with the leptin receptor (OB-R) is also depicted (**D**). Each mechanism ultimately leads to the transcription of target genes that promote breast cancer cell growth. ERα, estrogen receptor alpha; IGF-1R, insulin-like growth factor 1 receptor.

However, previous studies have demonstrated that ERα, in addition to its canonical genomic signaling pathway, is active outside the nucleus. Over the past decade, a number of researchers have successfully characterized several interactions between ERα and other signaling molecules that occur in the cytoplasm. For example, Song *et al*. [43,44] discovered that, in the presence of estradiol, ERα undergoes translocation to the plasma membrane and complexes with IGF-1R and the adaptor protein Shc, resulting in MAPK pathway activation. Down-regulation of IGF-1R prevents ERα translocation to the membrane, suggesting that IGF-1R signaling is necessary for nongenomic ERα activity. Ligand-bound ERα can also directly bind Src as well as p85, the regulatory subunit of PI3K, resulting in Akt activation downstream [45,46]. In addition, p85 can bind IGF-1R, leading to speculation that ERα may complex with both of these molecules upon activation by estradiol [47,48]. The receptor for leptin, an obesity-associated adipokine that was significantly elevated in our obese patient group (Table 1), has also been shown to crosstalk with IGF-1R, resulting in greater IGF-1R activation and an upregulation of Akt and ERK1/2 phosphorylation [49]. This interaction could potentially enhance IGF-1R/ERα crosstalk. Activated Akt and ERK1/2 can in turn activate ERα via phosphorylation at serine 167 and 118, respectively, within the receptor's AF-1 domain, leading to enhanced genomic ERα activity [50,51]. Figure 6 summarizes the different mechanisms of ERα activity. Because PI3K/Akt, MAPK and IGF-1R activity were all upregulated with obese patient sera exposure, we next explored the effects of obesity-associated factors on nongenomic ERα activity.

To determine whether obese patient sera promotes this nongenomic ERα activity and cross-talk with growth factor signaling pathways, we first examined the contribution of the PI3K/Akt, MAPK, and ERα pathways to obese patient sera-induced breast cancer cell viability and growth. Intriguingly, we found that a combination of the PI3K inhibitor LY 294,002 (LY) with the ERα inhibitor tamoxifen (Tam) most effectively mitigated the pro-growth effects of obese patient sera in the MCF-7 cells. The combination of PD 98,059 (PD) and Tam also demonstrated an attenuating effect on MCF-7 cell growth, so we were surprised that PD treatment alone stimulated significantly more cell growth than sera alone (Figure 4). This may be due to feedback upregulation of the PI3K/Akt pathway in response to MEK inhibition, as Hoeflich *et al*. [52] has demonstrated that the selective MEK inhibitor PD0325901 enhances PI3K/Akt signaling in several breast cancer cell lines. Together, these data support the possibility that crosstalk between both the PI3K/Akt and MAPK pathways and nongenomic ERα signaling may be playing a role in obesity-induced postmenopausal breast cancer progression, although the PI3K/Akt pathway may be the more important mediator of these effects. Additional evidence to support this conclusion includes the observation that Tam alone is sufficient to decrease obese patient sera-induced Akt and ERK1/2 activation to the levels observed in breast cancer cells grown in control patient serum (Figure 5).

In addition to demonstrating that obesity-associated circulating factors increase ERα-mediated Akt and MAPK activation, we also found that they stimulated greater Akt-mediated phosphorylation of ERα at serine 167 in MCF-7 cells (Figure 5). In contrast, exposure to obese patient sera did not upregulate ERα phosphorylation at the MAPK target site (serine 118), but researchers have found that breast cancer cell MAPK activity does not always correlate with phosphorylation at this site [53]. This ligand-independent activation of ERα via its AF-1 domain is a purported mechanism by which endocrine resistance can develop [18,19]. However, ligand-independent ERα activity is thought to be limited to the nucleus, where phosphorylated ERα acts as a transcription factor or co-factor (Figure 6B). As we did not detect a difference in ERα genomic activity, it is unclear whether the obese patient sera-induced increase in pERα(s167) has any biological significance.

Given the lack of any detectable effect on genomic ERα activity, it is possible that the obese sera-induced breast cancer cell viability and growth may be independent of circulating estrogen levels. If this hypothesis is confirmed, it would suggest one mechanism by which obesity may contribute to the development of resistance to aromatase inhibitor therapy, a finding with potential clinical implications. This conjecture, as well as the proposed importance of the PI3K/Akt/mTOR pathway in mediating the effects of obesity-associated systemic factors, is supported by the literature on endocrine resistance. For example, Miller *et al*. [54] found that induction of hormone independence via long-term estrogen deprivation of ERα positive breast cancer cells was accompanied by an amplification of PI3K/Akt/mTor signaling linked to upstream IGF-1R/insulin receptor hyperactivation, similar to the effects of obese patient sera exposure. PI3K signaling was required for the induction of hormone independence, illustrating the key role this pathway plays in the development of endocrine resistance. An earlier study by Beeram *et al*. [55] demonstrated that MCF-7 cells expressing a constitutively active Akt were refractory to treatment with letrozole, fulvestrant and tamoxifen, providing further basis for our conclusions. Results indicated that the Akt-induced resistance was mediated by both ERα-dependent and independent mechanisms and that response to endocrine therapy in these cells was achieved only by combining letrozole with the mTOR inhibitor RAD001. Similarly, Cavazzoni *et al*. [56] found that letrozole-resistant, aromatase-overexpressing MCF-7/AROM cells displayed greater PI3K/Akt/mTOR and MAPK pathway activity. Further, mTOR inhibition with RAD001 was able to completely inhibit proliferation in this cell line. The authors correlated these results with an analysis of pathway activation in breast cancer patients who had progressed on letrozole, finding an upregulation of PI3KA, pAkt and p-mTOR after three months on treatment in comparison to the patients' pre-treatment baseline. All of these studies suggest that the PI3K/Akt/mTOR pathway and its interaction with ERα are key mediators in the development of resistance to aromatase inhibitors. Consequently, it is probable that an upregulation of the crosstalk between these pathways, as seen in ERα positive breast cancer cells grown in obese patient sera, will lead to aromatase inhibitor resistance and disease progression.

## Conclusions

The continuous rise in obesity rates around the world underscores the importance of identifying the molecular pathways by which obesity contributes to the pathogenesis and progression of numerous chronic diseases, including breast cancer. This study provides evidence that postmenopausal obesity enhances ERα positive breast cancer cell viability and growth via crosstalk between the ERα, PI3K/Akt and MAPK signaling pathways, suggesting that the addition of a PI3K/Akt/mTOR pathway inhibitor to aromatase inhibitor therapy may improve the clinical outcome of obese postmenopausal patients. Additional clarification of the crosstalk mechanisms responsible for the effects of obesity on postmenopausal breast cancer progression will be the goal of future studies.

## Abbreviations

AI: aromatase inhibitor; BMI: body mass index; DMSO: dimethyl sulfoxide; ERα: estrogen receptor alpha; ERE: estrogen response element; FBS: fetal bovine serum; IGFBP-1: insulin-like growth factor binding protein 1; IGF-1R: insulin-like growth factor receptor 1; IL: interleukin; IMEM: improved minimal essential medium; IRB: Institutional Review Board; MTT reagent: 3-(4,5-dmethylthiazol-2-yl)-2,5-diphenyltetrazolium bromide; PBS: phosphate buffered saline; PCR: polymerase chain reaction; PI3K: phosphatidylinositol-3-OH kinase; SFM: serum-free media; TNF: tumor necrosis factor; UTHSCSA: University of Texas Health Science Center at San Antonio.

## Competing interests

The authors declare that they have no competing interests.

## Authors' contributions

LB performed the MTT, colony formation and ERE luciferase assays as well as the qPCR and western blot analysis and drafted the manuscript. DC assisted with the MTT assays and western blot analysis and helped draft the manuscript. IM collected the patient characteristics from their medical records. AB collected the patient serum and helped draft the manuscript. SH participated in the design of the study and helped draft the manuscript. LD conceived of the study, participated in its design and coordination, and helped draft the manuscript. All authors read and approved the final manuscript.
